# Perception of Graphical Virtual Environments by Blind Users via Sensory Substitution

**DOI:** 10.1371/journal.pone.0147501

**Published:** 2016-02-16

**Authors:** Shachar Maidenbaum, Galit Buchs, Sami Abboud, Ori Lavi-Rotbain, Amir Amedi

**Affiliations:** 1 The Edmond and Lily Safra Center for Brain Research, Hebrew University of Jerusalem, Jerusalem, Israel; 2 Department of Medical Neurobiology, Institute for Medical Research Israel-Canada, Faculty of Medicine, Hebrew University of Jerusalem, Jerusalem, Israel; 3 Department of Cognitive Science, Faculty of Humanities, Hebrew University of Jerusalem, Jerusalem, Israel; 4 Sorbonne Universités UPMC Univ Paris 06, Institut de la Vision Paris, Paris, France; Emory University, UNITED STATES

## Abstract

Graphical virtual environments are currently far from accessible to blind users as their content is mostly visual. This is especially unfortunate as these environments hold great potential for this population for purposes such as safe orientation, education, and entertainment. Previous tools have increased accessibility but there is still a long way to go. Visual-to-audio Sensory-Substitution-Devices (SSDs) can increase accessibility generically by sonifying on-screen content regardless of the specific environment and offer increased accessibility without the use of expensive dedicated peripherals like electrode/vibrator arrays. Using SSDs virtually utilizes similar skills as when using them in the real world, enabling both training on the device and training on environments virtually before real-world visits. This could enable more complex, standardized and autonomous SSD training and new insights into multisensory interaction and the visually-deprived brain. However, whether congenitally blind users, who have never experienced virtual environments, will be able to use this information for successful perception and interaction within them is currently unclear.We tested this using the EyeMusic SSD, which conveys whole-scene visual information, to perform virtual tasks otherwise impossible without vision. Congenitally blind users had to navigate virtual environments and find doors, differentiate between them based on their features (Experiment1:task1) and surroundings (Experiment1:task2) and walk through them; these tasks were accomplished with a 95% and 97% success rate, respectively. We further explored the reactions of congenitally blind users during their first interaction with a more complex virtual environment than in the previous tasks–walking down a virtual street, recognizing different features of houses and trees, navigating to cross-walks, etc. Users reacted enthusiastically and reported feeling immersed within the environment. They highlighted the potential usefulness of such environments for understanding what visual scenes are supposed to look like and their potential for complex training and suggested many future environments they wished to experience.

## Introduction

Imagine yourself immersed in a virtual world. Navigating down a street to a place you have never been to and might soon want to visit, engaged in a first-person quest, or in the middle of a social event, when suddenly the screen turns off. Deprived of the visual information you are simply shut out of these virtual worlds, which are currently mainly based on visual cues.

While to most users such a problem is rare, for over 39 million blind people living worldwide [1] these environments are simply not adequately accessible.

This is especially unfortunate as virtual worlds hold great potential for these users. For example, since the transfer of orientation and mobility knowledge between virtual and real environments, at least to a certain extent, is well established, including for users who are blind [2–6], these worlds can be used for familiarization with an unknown environment virtually before going there physically, e.g., via Google-Street-view. This has the benefit of familiarizing them with the environment while avoiding the risks associated with such exploration in the real world like injuries [7] or getting lost [8]. This is especially useful as this can be done not only with a personal orientation and mobility trainer as done today, but also alone between sessions with one's trainer at one's own leisure, decreasing the cost and availability problems of personal trainers [9] in parallel to boosting the effectiveness of sessions with said trainers, and ultimately increasing independence. Similarly, these environments hold the potential to be used safely for a wide range of additional purposes in and of themselves ranging from education and scientific research to social interaction and games. Within the general goal of increasing accessibility to disabled individuals (even by law in some countries) and the current predictions of bigger dependency on such environments for work and social life, this issue is becoming more and more important.

This challenge is not new, and there have been several previous efforts to overcome it. One direction has been to try and use existing accessibility tools for blind individuals, typically screen readers or Braille displays [10]. Unfortunately, these are not adequate for the task as they are mainly text-based and do not process or present graphical information. A second approach has been the creation of new dedicated environments aimed at these users which are accessible to them, for example by having all objects within them clearly tagged and with tools for proper parsing between these tags in every scene (see for example [9,11–13]). While there are several very successful examples to this approach, their use still remains limited and it does not provide access to mainstream virtual environments. A third option has been creating dedicated environment-specific accessibility tools for existing mainstream environments, such as creating a virtual white-cane or virtual guide dog within Second-Life which are inherently part of the environment and can interact fully within it (see examples of this approach reviewed in [12,13]). Both the second and third approaches include the addition of redundant non-visual information within these environments (such as unique auditory or vibro-tactile cues) which does not interfere with the experience of sighted users, an approach which has already seen some success in increasing the accessibility of 2D graphical information [14–16].

These tools have suffered from problems with the labour-intensive and high pre-processing requirements of making the environments work with them and the reliance on the creators of the original content to properly make their content accessible. Another problem with these approaches is that they often require an environment-specific learning of the sensory transformation, and as this transformation is not useful outside of the specific environment, and especially not in the real world, it limits the motivation and ability to learn them.

Thus, current tools offer solutions to some use cases, but there is still a long way to go in making these environments properly accessible and fully perceptible to blind users.

We suggest here the use of Sensory Substitution Devices (SSDs) as another complementary tool for this purpose [17–20]. SSDs are non-invasive interfaces which translate information from one sense into another, here from vision into audition [21]. These devices have been used extensively for psychophysical [17,19,22,23] and neuroimaging [24–27] research and to a more limited extent also for practical use in the real world [28,29]. Their transfer to practical use still faces some significant challenges, and though many of these have been mitigated in recent years one of the main remaining challenges is in training and mastering their use (reviewed at length in [30,31]). Specifically here, for the purpose of this study, we used the well-established EyeMusic SSD [32], a visual-to-auditory SSD which transforms the visual parameters of shape, location, brightness and color into sound from the entire scene.

The main advantages of using SSDs are that they use the exact same transformation for these virtual tasks as for any other task in the real world. Thus, users can capitalize on experience and skills from general use of the device for increasing the accessibility of the virtual environments as a goal in and of themselves for purposes such as education and games. Furthermore, this enables their potential use for training both to master using the device's transformation, for learning specific real world environments and for learning complex visual principles, such as the changes in a first person view of a visual scene while moving through it [30,31]. Importantly, learning real-world environments in this way allows isolating the learning process of the visual information from other challenges in the real world, such as aiming the camera while moving, and focusing on separate skills which might help ease the users' transition into real world use.

Another advantage in using SSDs is that they can convey the raw visual information displayed on the screen, and thus do not require any code interface to the target environment or pre-processing within it, leading to genericity across any virtual environment and independence from relying on the original contents’ creator. The information that is sonified is exactly the same as a sighted user would see on the screen!

Previous steps in the direction described in this work tried to use SSDs within the framework of the other options mentioned above without taking advantage of this ability–using them as in-world tools, requiring environment-specific dedicated programing. This included navigation with BrainPort in simple routes [27,33] and our own use of a virtual version of the EyeCane [34,35] [36–38].

Here, we test the use of visual-to-auditory SSDs for virtual world perception by giving blind participants, who had extensive previous experience with the EyeMusic, several tasks in simple environments with increasing complexity in which they were required to recognize goals and navigate correctly to them, tasks normally impossible in virtual environments without vision ("**Experiment 1**").

**Experiment1: Task 1** required locating and then recognizing 3 doors, differentiating between them and explaining the difference, and then finally walking through the one which was different (e.g., in shape or in color) from the two others. In **Experiment1: Task 2**, the choice of which door to navigate through was based on the door's surroundings (e.g., the target door is between a house and a tree), showing contextual understanding of the virtual environment. These tasks required the users to not only understand what objects were in the environment but also their relative spatial location and their changes while moving towards, and eventually through, them.

In **Experiment1: Task 3** blind users were exposed to a more complex virtual environment in which they performed a series of uncontrolled and unstructured perceptual tasks, enabling us to gather their general perception of the environment and their reaction to it.

We used these tasks to test two types of questions:

1. Could congenitally blind users, who never experienced any virtual environments at all, perceive them and perform tasks in them?
2. How did these users feel about these environments? Did they feel immersed within them?

Additionally, we conducted a separate additional experiment ("**experiment 2**") to explore the difference in non-visual interaction with these environments when users were not offered any assistive device compared to when they could use the EyeMusic after minimal training (<20m). For the purpose of this comparison two groups of sighted-blindfolded participants, with and without using the EyeMusic, performed Experiment2:task 1.

We found that despite their initial hesitations and disbelief the congenitally blind participants of experiment 1 could perform these three tasks with a very high success rate and reported a high feeling of immersion in the virtual worlds. We additionally confirmed with experiment 2 that the group of sighted-blindfolded without the EyeMusic could not perform the tasks even at chance level but that those who had just a short training session could, to a certain extent, complete Experiment1:Task 1 successfully.

## Methods

### Sensory substitution, EyeMusic and their use in this study

#### 1.1.1 Sensory Substitution

The approach we suggest here uses a class of tools known as Sensory Substitution Devices (SSDs) [18,30], in which the visual information in the scene is transferred to the user via different senses. SSDs rely on the ability of the brain to interpret this information coming through a different sense and extract the relevant information from it [19,30]. Importantly, this approach is distinct from one which processes the scene and describes it to the user, as it offers the user low-level perceptual sensory information which he must then process. Users at first have to focus and explicitly translate this information, but as they gain experience the process becomes more and more automatic, and some late blind users report their sensation as becoming similar to ‘seeing’, for instance, an image of a cup popping up in their mind's eye when they look and interact with the cup via the SSD [29]. These devices have been used over the past decade for a wide variety of tasks, including in noisy, cluttered real-world environments, such as identifying objects, locating them and reaching for them (see advanced user-studies in [28,29,39] and some structured examples at [30])

Despite their potential, SSDs have not been widely adopted. On the setup aspect, up until recently these devices were not practical to use due to weight, cost, cumbersomeness, and unavailability. These factors have been significantly mitigated by the current prevalence of smartphones enabling SSDs such as the vOICe [21] or EyeMusic [32] to be freely available for download with no requirements for additional hardware peripherals such as expensive electrode or tactile arrays. On the transformation front, recent decades have seen a flourishing of multimodal and cross-modal research, which combined with new computer vision algorithms and new sensors such as depth cameras enable SSDs to deliver much better visual information to the user.

The main stumbling block still in the path of the adoption of SSDs is their learning process. SSDs require long training protocols, which do not exist for most devices. Even for those few devices where they are available, the assistance of a sighted friend or instructor is required for all stages of the training. In many devices, especially those relying on auditory transformations, it takes quite a while until the SSD can be used for practical tasks. Many, if not most, SSD users quit in frustration after several attempts to use the devices [19,30]. Several teams worldwide are working on upgrading this experience by gamifying the training process, speeding up the learning or creating automated standardized training protocols [30].

#### 1.1.2 The EyeMusic

**Fig 1:**
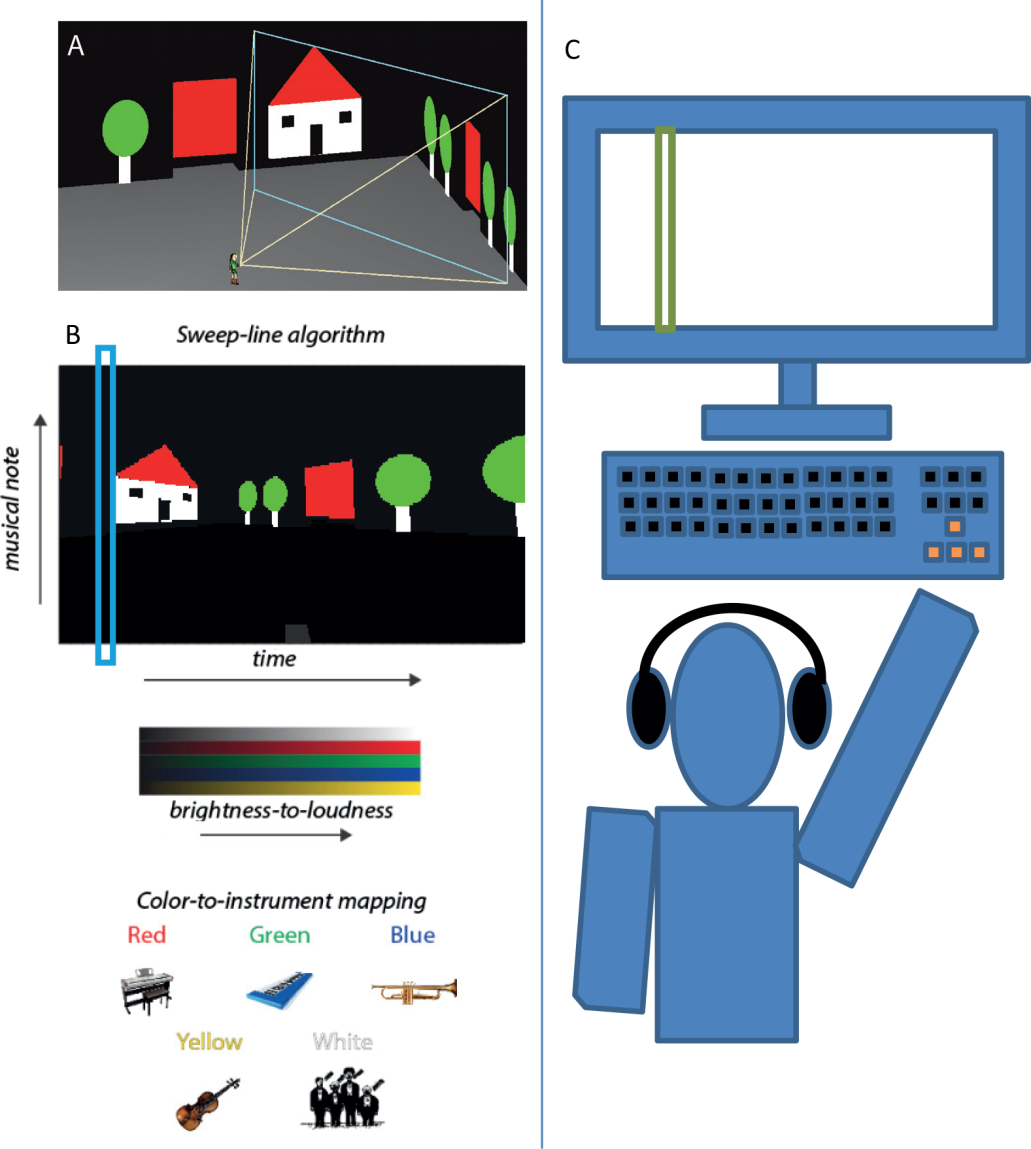
The EyeMusic SSD and experimental setup. (A) A 3^rd^ person view of the user's avatar and field of view, the contents of which will be sonified in the EyeMusic's algorithm shown below it. (B) Illustrates the principles of the EyeMusic visual-to-audio transformation: after down-sampling images to 24*40, each pixel in the image is assigned a value such that its y-axis value is converted to a musical note (the higher the value the higher the note), its x-axis value to time (the closer to the left the earlier in the soundscape), its brightness to volume and its color to musical instrument (C) Illustration of the experimental setup.

The EyeMusic (Fig 1) is a visual-to-auditory SSD, described in detail in [32], and used for various real-world and experimental tasks [40,41]. It is also freely available as an iOS App (http://tinyurl.com/oe8d4p4) and for Android on Google Play (http://tinyurl.com/on6lz4e).

The EyeMusic can receive an image from a file or camera, or as in the case of this experiment from a computer via sequential screen-shots, capturing all of the visual information on the screen. This image is segmented in turn to a 24*40 matrix, and each cell is assigned a value by its location, color, and brightness. The Hue, Saturation and Lightness (HSL) values are clustered into 6 colors, 5 of which are mapped to musical instruments (red, green, blue, yellow & white) and black which is silent. Brightness is represented by volume. The location is represented by a rising blues scale of musical notes such that the higher the Y value is, the higher the note. The X axis is converted into time within the sonification–i.e. when played, the image will sound the columns in a sweep line from left to right, similar to the sonification scheme established by the veteran vOICe SSD [21] or the Vox [42].

For example, a white horizontal line in the top left corner of the screen will result in a sonification of a continuous chorus on a high note followed by silence. Making the line lower would make the note lower. Moving the line to the right would add silence before the line. Changing the line's color from white to blue would change the musical instrument from a chorus to a trumpet. We recently gave a TEDx talk in which several examples of the EyeMusic soundscapes can be found (http://www.youtube.com/watch?v=jVBp2nDmg7E).

For a more detailed explanation about the transformation see [32]. The algorithm is illustrated in Fig 1.

The default EyeMusic frame-rate is one-per-3-seconds. In these experiments the frame-rate was freely controlled by the users whose preferred rate varied both between users and throughout the experiment, but was typically at one-per-2-seconds.

In this research we used the EyeMusic SSD, which is only one of many auditory-to-visual SSDs currently available. Others include the vOICe, which has the largest user community, the Vibe [43], and many others (see review [19,30]). We do not claim here any specific advantages to the EyeMusic and indeed suggest our results can be generalized for visual-to-auditory SSDs in general, as long as they have the minimum requirements for interaction with the environment (chiefly a screen sonification mode and the ability to convey colors). Potentially, these results might also generalize to visual-to-tactile SSDs as well (though with the additional requirement of their dedicated hardware).

#### 1.1.3 The sonification process

In these experiments EyeMusic was run as software on the same computer the experiment was run on, similar to other accessibility tools such as screen-readers, and not from the smartphone setup mentioned above. At every scan, typically 1 per 2 seconds but dynamically controlled by the user, EyeMusic sampled the graphical information via a screenshot of the contents displayed on the screen and sonified it for the user. As the user moved in the virtual environment via the keyboard the contents of their surroundings, and correspondingly the information displayed on the screen unseen by the users, changed according to standard 1^st^ person view, and thus the next round of screen sonification matched the current viewpoint. The users accessed this information via standard headphones, without requiring any additional peripherals.

### Experimental setup

#### 1.1.4 Equipment and setup

The virtual environments in this experiment were created using Blender 2.49, and Python 2.6.2. Participants were seated comfortably in front of a computer. Navigation was accomplished using the arrow keys on a standard keyboard ("up" arrow for moving 0.4 m forward, "left/right" arrows for rotating the avatar 30 degrees left/right accordingly), and the auditory cues were delivered via standard headphones (see Fig 1C). The environments were scaled such that each "virtual meter" matched a real world meter. The sighted participants of experiment 2 were blindfolded throughout the experiment using Mindfold blindfolds.

#### 1.1.4.1 Experiment 1 participants

The main experimental group consisted of eight congenitally blind participants who took part in Experiment1:tasks 1–2 and seven congenitally blind participants who took part in Experiment1:task 3. Participants were designated A1-9. Note that participants A4 and A5 only took part in Experiment1:tasks 1–2 and participant A6 only took part in Experiment1:task 3 (see Table 1 for full details on the participants and the tasks they took part in).

**Table 1:** Information on the congenitally blind participants of experiment 1.

Code	Age	Age of blindness	Reason of blindness	Residual vision	Navigation aids in use	Participation in Experiment1:tasks 1–2	Participation in Experiment1:task 3
**A1**	33	Birth	Anophthalmia	None	Cane+Dog	Yes	Yes
**A2**	45	Birth	Excess Oxygen at birth	None	Cane	Yes	Yes
**A3**	33	Birth	Unknown	Faint light/dark perception	Cane+Dog	Yes	Yes
**A4**	40	Birth	Anophthalmia	None	Cane	Yes	No
**A5**	43	Birth	Anophthalmia	None	Cane	Yes	No
**A6**	42	Birth	Lieber	Faint light/dark perception	Cane	No	Yes
**A7**	40	Birth	Microphthalmus	Faint light/dark perception in right eye	Cane+Dog	Yes	Yes
**A8**	42	Birth	Pre-birth Rubella	None	Cane	Yes	Yes
**A9**	39	Birth	Excess oxygen at birth	None	Cane	Yes	Yes

All blind users were independent navigators, with experience in the use of a white-cane (100%) and several in the use of a guide dog as well (33%). Participants reported being familiar with GPS devices and experiencing them, but none made practical use of them or had undergone training with them.

None of the blind participants had ever been previously exposed to virtual environments. All had extensive experience using the EyeMusic with static stimuli and limited experience using it in simple 2D games and in the real world as part of previous structured training in our lab (for example see [28,32,40]). This previous structured training consisted of weekly meeting with a personal instructor from the lab, similar to those we conducted with the vOICe SSD as described in [24,25], and were part of a general rehabilitation program not dedicated for this experiment leading to significant differences in experience between participants.

#### 1.1.4.2 Experiment 2 Participants

A separate experiment exploring the EyeMusic in virtual environments included two groups of sighted-blindfolded users who performed **Experiment2:**task 1 (identical to **Experiment1:task1)** in a device "on" vs. "off" paradigm to explore the EyeMusic's effect. They consisted of ten sighted-blindfolded participants (9 female, 9 right-handed, age ranged 21–28, designated C101-C110) who did not use any assistive technology, simulating the experience of a blind user attempting this task without any graphical accessibility tools, and 19 sighted-blindfolded participants (11 female, all right-handed, age ranged 21–28, designated C201-C219) who used the EyeMusic after only minimal training (<20 min).

Note that the performance of the Experiment 2 groups were **not** compared to that of the blind group of Experiment 1, but rather were used to explore different secondary points (i.e. can these tasks be performed without the device? What is the effect of having the device on-off? What was their level of immersion?).

#### 1.1.5 Ethics and language

This experiment was approved by the Hebrew University Ethics Committee, and all participants signed informed consent forms. As all experiments were conducted in Hebrew, user quotes were translated to English by the authors during manuscript preparation.

### Experimental design

#### 1.1.6 Training for tasks 1& 2

The blind participants of experiment 1 underwent a short EyeMusic warm-up (see images in S1 Fig) and received some dedicated training for these environments (<10 minutes)—mainly explaining the concept of virtual environments and going over the keyboard controls and over a list of relevant visual principles (e.g. "as you approach an object it's visual representation on the screen will grow", "when you turn to the left an object that was in the center of your view will now be on the right" etc.–see S1 File). Users were told that they could request the frame-rate to be increased or decreased at any time and different speeds were demonstrated (default was set to 1 frame per 2 seconds; typically participants did not make any changes). Since the experiments sought to explore the users' first exposure to the virtual environments, participants did not receive any training experience with the environments themselves, and the experiment was started as soon as the participants said they felt ready to begin.

#### 1.1.7 Task 1 (both experiments 1 & 2)

**Fig 2:**
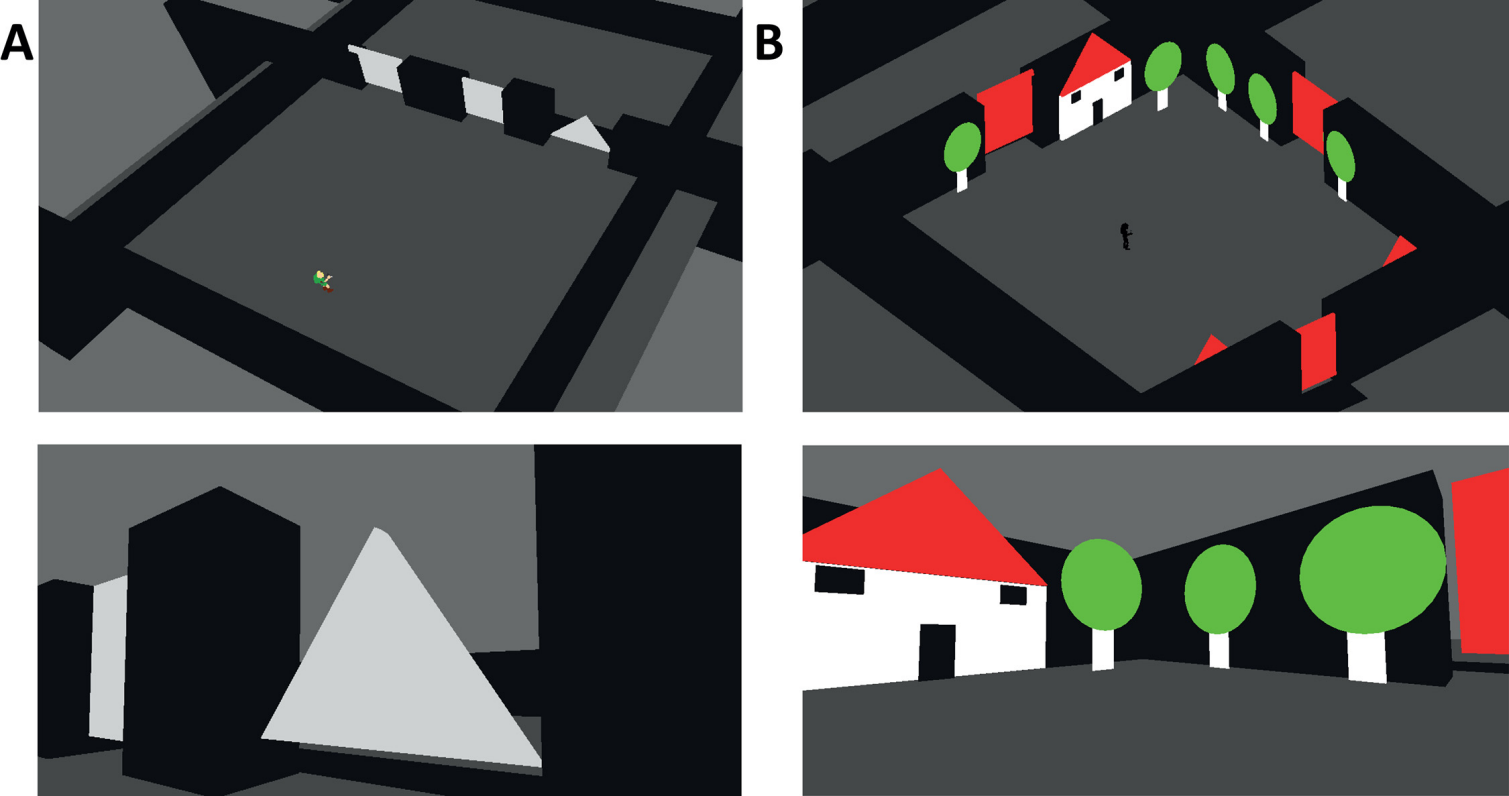
Screenshots from tasks 1–2. (A) Two screenshots from Task 1, from above demonstrating the level's layout (top) and from the user's 1^st^ person view demonstrating a triangular door and a square door seen at an angle (bottom) (B) Two screenshots from Experiment1:Task 2, from above demonstrating the level's layout (top) and from the users 1^st^ person view demonstrating trees, a house and a door (bottom).

This task included 12 pseudo-randomly ordered trials. Each trial consisted of a virtual environment with 3 doors in front of the user. The environment was a simulated room and the whole space was navigable. The doors were situated 16m to 18.5m away, translating into a minimum of ~45 key presses for an optimal path. In each trial, one of the three doors differed in shape or color (Fig 2A). Participants could not fully see all three doors from their starting position, nor could they all be fully caught in a single frame, and thus the participants had to turn or move to see them all. A maximum time limit of 7 minutes was set per trial. Participants had to perform three assignments:

1. Determine which door was different (left/middle/right).
2. Explain verbally in what aspect it was different.
3. Navigate correctly to that door and complete the trial by exiting through it.

Accordingly, a conservative 1/3 was used as a chance level for statistical purposes (though chance performance in navigation, in which the user would not necessarily reach any door, is likely far lower as demonstrated by the "device off" group of experiment 2).

Users did not receive any feedback throughout this task.

Following the task, users were asked to report their level of immersion (no/somewhat/yes) and to supply general feedback on the task (difficulty, enjoyment, general thoughts, etc.).

#### 1.1.8 Task 2

This task included 6 trials. Each trial consisted of a virtual environment with 3 doors in various locations around the user who is standing at the center of the virtual environment (Fig 2B). The environment simulated an outdoor space and the whole space was navigable. The environment also included houses and trees such that in each trial one door was between trees, the second between houses, and the third between a house and a tree (Fig 2B). There were also several additional trees and houses which were not adjacent to a door. The doors were 18m away in different orientations, translating into a minimum of ~45 key presses for an optimal path. Participants could not fully see all three doors from their starting position, nor could even 2 doors be caught in a single frame, and thus they had to turn or move to see them all. The environment was the same across trials but the user's starting orientation varied. A maximum time limit of 7 minutes was set per trial. Participants were given the description of the goal door's surroundings (e.g. "find the door with trees on both sides"), and then had to perform two assignments:

1. Determine which door is their target.
2. Navigate correctly through it.

Accordingly, a conservative 1/3 was used as a chance level for statistical purposes (though chance performance in navigation, in which the user would not necessarily reach any doors, is likely far lower).

Users did not receive any feedback throughout this task.

Following the task, users were asked to report their level of immersion (no/somewhat/yes) and to supply general feedback on the task (difficulty, enjoyment, general thoughts, etc.).

#### 1.1.9 Experiment1:Task 3

**Fig 3:**
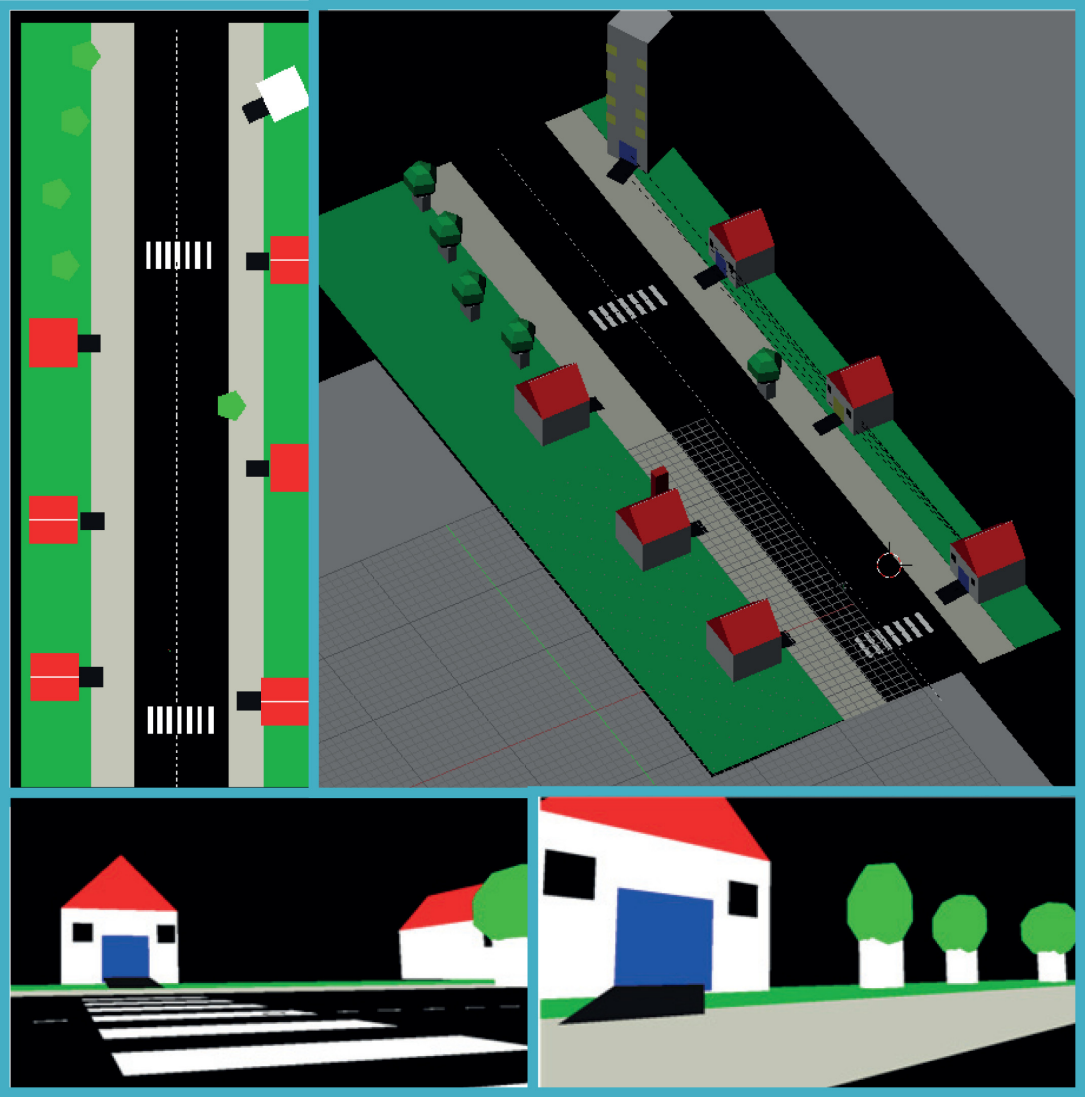
Screenshots from Experiment1:Task 3. Including views from directly above (top left), from above at an angle (top right) and screenshots to be sonified from the user's 1^st^ person perspective showing houses, trees and a crosswalk (bottom).

The third task was an exposure to a more complex virtual environment, though still far from realistic or from mainstream virtual worlds. Participants were asked to freely interact with it and perform a fluid set of tasks (see Fig 3 for screenshots and overhead view). The environment was a simulated street, including trees and houses in different sizes and shapes, sidewalks, lawns and a road with crosswalks and marked lanes. The whole space was navigable. It should be emphasized that this task was not a controlled experiment but rather a semi-structured experience in which user and instructor explored the environments together. Participants were free to ask the instructor questions throughout the navigation. Exposure to the environments lasted until participants requested to stop.

During the task all participants were asked to:

1. Recognize a house from several different angles.
2. Explain the difference between different houses (window color and number, chimney etc.) and navigate to specific ones.
3. Recognize trees and count them.
4. Describe their environment.
5. Recognize the crosswalkand cross the street on it.

Additionally, the instructor repeatedly asked the participant to describe what he/she sees, and when participants pointed out a feature of interest encouraged them to go and explore it.

Following the experiment, participants were asked the following questions:

1. How difficult was this task? (1–5)
2. How interesting was the task? (1–5)
3. Did you feel immersed in the environment? (no/somewhat/yes)
4. Do you see any future potential for you with it? (free text)
5. What were the most interesting experiences you remember from the virtual environment? (free text)
6. Were there things you were surprised to discover? (free text)

### Statistics

P-values were calculated using Matlab's non-parametric Wilcoxon Rank-sum (unsigned) function. Note that since this test offers a discrete set of possible p-value results, all of the statistical tests which had a maximal success score received the same identical optimal p-value (i.e. after correction for multiple comparisons p<1.5*10^4 for the blind participants in experiment1:Tasks 1–2 and p<9.8*10^-6 for experiment2:Task1). Results are presented in the format of <Mean>±<Standard Deviation>.

## Results

### 1.1.10 Experiment 1:Tasks 1–2—blind users

**Fig 4:**
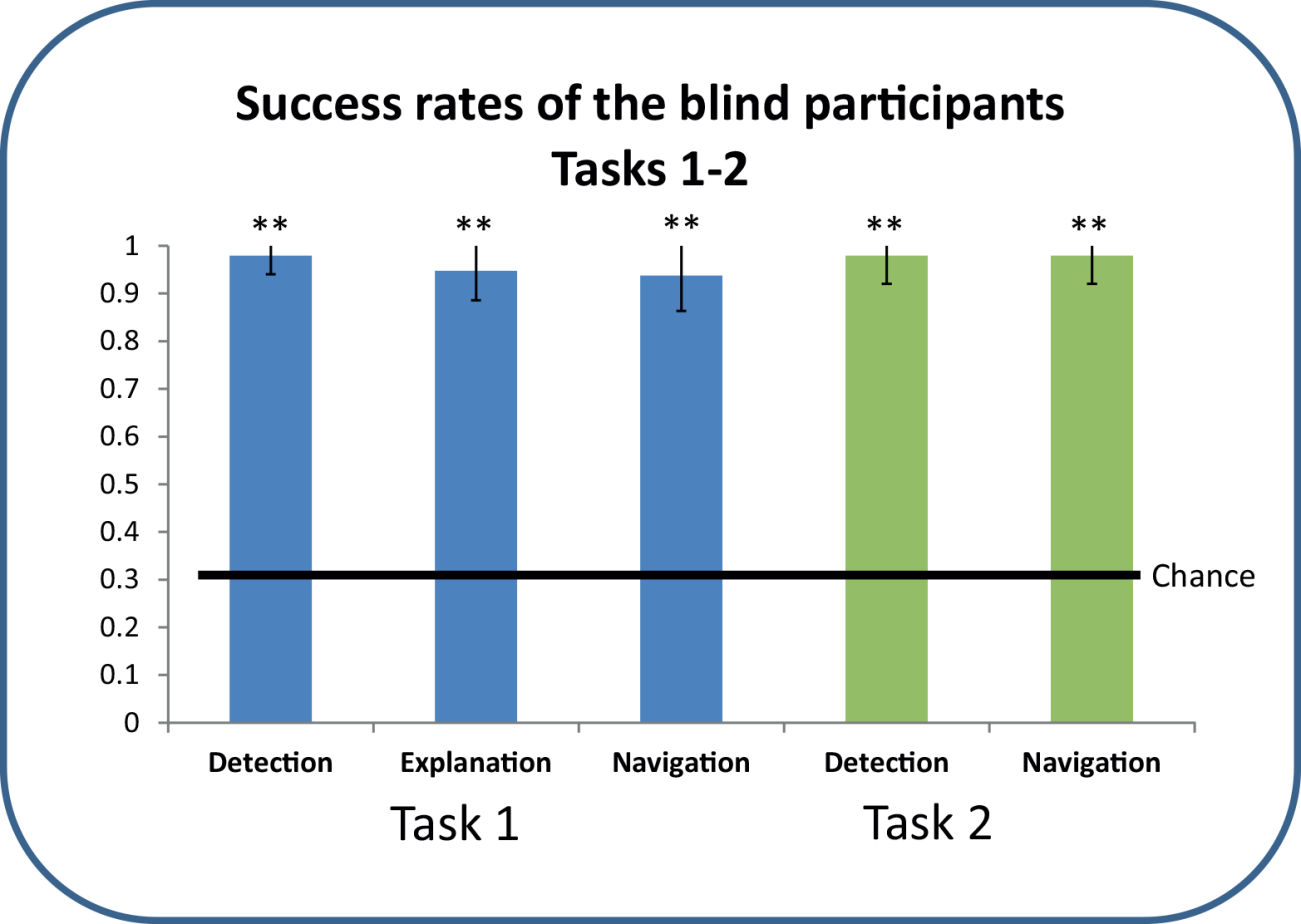
Results of the two tasks. Scores for Detection of the different door, Explaining why it is different and Navigating to it correctly in Experiment1:Task 1 (blue), and for Detection of the correct door and navigating through it in Experiment1:Task 2 (green). Error bars represent SD.

The blind participants were easily able to complete all three parts of Experiment1:task 1, with a success rate of 97.9±3.8% (p<1.5*10^4, compared to chance) for recognition of which door was different, 94.7±6.2% (p<1.5*10^4, compared to chance) for correctly explaining how it was different, and 93.7±7.3% (p<1.5*10^4, compared to chance) for navigating correctly to the exit (See Fig 4). Average time per trial was 86.9±19.3s.

They were also easily able to complete both parts of Experiment1:Task 2, with a success rate of 97.9±5.8% (p<1.5*10^4, compared to chance) for recognition of the correct door and 97.9±5.8% (p<1.5*10^4, compared to chance) for navigating correctly to it (See Fig 4). Average time per trial was 119.1±28s.

Before the tasks several participants reported having trouble conceptualizing the idea of a virtual world and especially the idea of viewing it from 1^st^ person perspective.

After completing the tasks the participants reported that the tasks had been interesting and fun and that they had experienced a feeling of immersion within them, with six participants answering they "felt immersed" and the seventh and eighth answering "felt somewhat immersed" or in the words of one of these participants "*now I know what you meant*, *it was like I was really there and the objects were all around me*" (participant A3). They focused their remarks both on the potential application of such environments to their lives, specifically for purposes of navigation, and also on the potential of this approach for increasing the accessibility of games. They also reported that their main difficulty was the need to remember and utilize the visual principles (e.g., an object grows larger as you move closer to it–"*It is just so unintuitive that to be able to see the top of the tree and not just the trunk I have to walk further away from it*. *Usually if I want to sense something better I need to get closer to touch it or listen to it*" (participant A7)). All participants agreed enthusiastically to participate in future experiments using this approach.

The participants noticed that the environment was the same in all trials, despite the differences in starting orientation, and took advantage of the learned spatial layout in subsequent trials "*I know where I am*, *there are three neighbouring houses here*, *if I walk and turn to the left I'll find the door which is between the houses and trees*" (participant A3). "*Hmm*, *the door between two trees*. *I remember that one*, *I need to find the place where there are four trees in a row*, *and it's to their left*" (participant A8).

### 1.1.11 Experiment 2: Sighted-blindfolded participants performing task 1

Note that these groups were not compared to the blind group in any way, due to the difference in experience with the device and familiarity with visual scenes and principles, and accordingly were compared only to each other.

**Fig 5:**
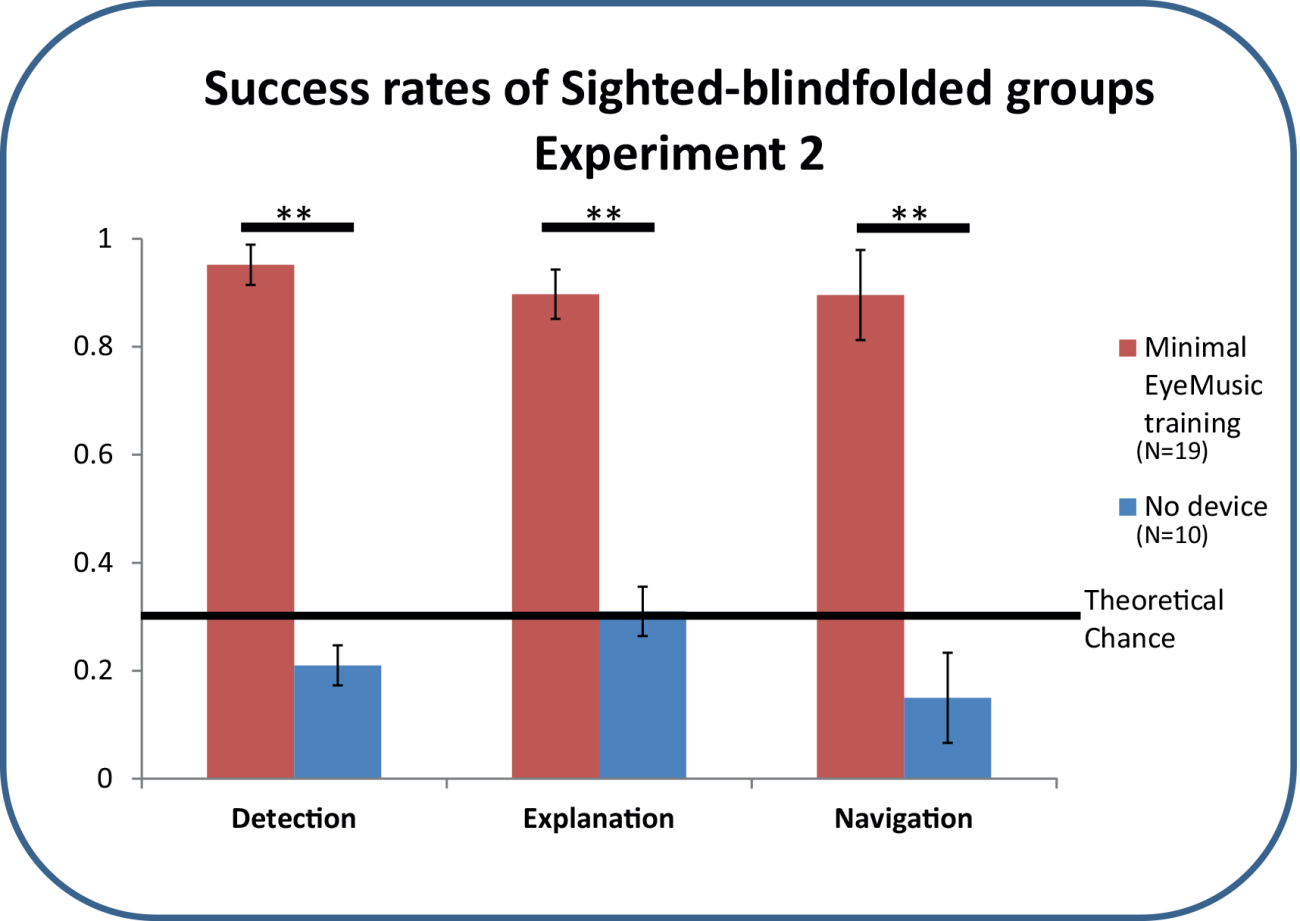
Results of the two sighted-blindfolded groups of Experiment 2. Detection of the different door, Explaining why it is different and Navigating to it correctly in Task 1. Error bars represent SD.

The Device-Off group, who performed this task without the EyeMusic or any other assistive device, failed at almost all trials with scores of 21±13% for recognition of which door was different, 31±11.9% for correctly explaining how it was different, and 15.5±14% for navigating correctly to the exit (Fig 5). Average time per trial was 82.4±14s.

This group reported great frustration–"*These tasks are impossible*! *What's the point*?! *How am I supposed to describe what's on the screen if I can't see it*?" (participant C105), "*I'm just guessing*. *I have no idea what I'm doing or where anything is*" (participant C8). Most participants refused to take part in future similar experiments "*Ok*, *Ok*, *I get it*, *not being able to see is really limiting and annoying*, *and I can sympathize better with how frustrating that is*. *But I think I've got the point enough to not want to have to go through that again*" (participant C107). All of these participants reported no immersion.

The Device-On group, who used the EyeMusic with minimal training were easily able to complete all three parts of the task, with a highly significant success rate of 95.1±8.1% (p<6.3*10^^-6^ in comparison to the Device-Off group) for recognition of which door was different, 89.7±11.9% (p<1.1*10^^-5^ in comparison to the Device-Off group) for correctly explaining how it was different, and 89.5±13% (p<9.8*10^^-6^ in comparison to the Device-Off group) for navigating correctly to the exit (See Fig 5).

These participants tended to describe the experiment as an interesting game and reported enjoying the tasks. They reported their main difficulty as being in extracting the visual information from the auditory cues. All of these participants agreed enthusiastically to participate in future experiments using this approach. 12 participants reported feeling immersed, 6 somewhat and 1 not at all.

### 1.1.12 Experiment1:Task 3 –Blind participants

It should be emphasized that navigation in the more complex virtual environment of this task was focused more on getting user reactions then on accomplishing specific tasks.

All participants were able to perceive the complex environment well enough to successfully perform the simple uncontrolled tasks described above. Participants spent 45–80 minutes within the environments.

Participants described the task as very difficult (8/8), but also as very interesting (8/8) and as something they wished to experience again and focus training on the relevant skills "*I can see the practical purpose here*. *It's hard*, *but if I learn to do it this can be a great tool*, *so it's worth it*" (participant A3). "*I feel like this won't only help me with your device since it teaches me what the real world is supposed to look like and introduces me to features I wasn't aware of*" (participant A1)).

Interestingly, several of the participants described their experience at first as "*moving the surrounding shapes around*" (participant A6), but then as they viewed more complex parts of the scene reported an increasing sense of immersion "*I'm not sure at what point it happened*, *but it switched*. *I think because of the complexity and the amount of information*. *Suddenly it's me moving through the shape and not the shapes being rotated and distorted*".

After completing the session all participants reported feeling immersed, or in the words of participant A7 "*It was like I was just in there*, *walking on the street next to the houses*". Some reported grounding themselves spatially with landmarks "*I could tell where I was in the street by the high-riser*, *and what was happening on the other side of the road*, *so I knew where to go to find the crosswalk*" (participant A8).

Of special interest were features that they could recognize themselves. For example, all 6 participants were very excited to be able to identify a crosswalk "*That ding-ding-ding is very distinctive from the right angle*. *This could be a very useful skill to have when walking around*. *Finding crosswalks on small streets often requires me to ask people*" (participant A1). When recognizing the 3D trees and houses in this environment several participants compared it to their experience in Task-2-like environments "*It's like the painting popped out*. *I can walk around it*" (participant A1). The reactions were even more pronounced for visual features they were not previously aware of "*I never knew there were lines between lanes in the road*, *nobody ever mentioned them and I never even thought to ask*" (participant A1).

Another interesting observation was the process of learning visual rules. For example, when asked to describe the part of the street in view, participant A3 at first described it as "*there are 3 houses on each side*, *a small*, *medium*, *and large one with the larger ones on the outside and the smaller ones closest to the road*". After being asked to walk towards them she suddenly said "*I can see the size change*! *This is just like what you taught me*, *the houses are actually probably all the same size but just at different distances*, *right*?" (participant A3). Another example was the effect of distance on the contents of the visual field "*The most interesting part to me was how when I stood right next to the wall I couldn't see the roof*. *The idea of moving further away to be able to see it was very unintuitive to me–I expected to see something better when I get closer like getting closer to touch or listen*. *But after the first few houses it suddenly started making sense*" (participant A2).

When several sighted-blindfolded participants from the device-off group from experiment 1 were asked to participate also in this task they reacted with bafflement. These participants typically quit the task within the first minute, thereby preventing any analysis of their experience and this resulted in exclusion from the rest of this project. "*You want me to do what*? *How am I supposed to walk towards a house or describe it to you if I can't see anything and don’t even know if there really is a house on the screen*?!" (participant C09).

## Discussion

These results provide a proof-of-concept that congenitally blind users can indeed perceive virtual environments and perform tasks in them, and that this can be done via SSDs, in this case via EyeMusic, and thus accomplish virtual tasks otherwise inaccessible to them. These include both scene comprehension and dynamic navigation to a target, and basic perception of a more complex scene.

### User Reactions

The blind participants noted that they currently have no other way of performing these sorts of tasks, and were enthusiastic about the potential, mainly for navigation training and for gaming (for example: *"Could you build me a model of my home*? *And of the way to your lab*?*"* (participant A6); *"Ok*, *now I want you to make the same game*, *just with monsters and shooting*, *and let me take it home with me"* (participant A7)).

A known problem with SSDs is the unpleasantness of their output. The participants’ reported enjoyment and their enthusiasm for future use of the approach indicate that users found the current version acceptable. This is especially true for the user reactions to the more complex environments, which despite their difficulty engendered high levels of motivation and immersion in the users.

An especially interesting reaction is the reported level of immersion by the congenitally blind users, as to the best of our knowledge this is the first report of such a sentiment, and it is not intuitive that immersion could have been reached non-visually.

The negative reactions from the control group demonstrate how frustrating the accessibility situation can be without assistive devices.

### Gamification and dynamic control

**Fig 6:**
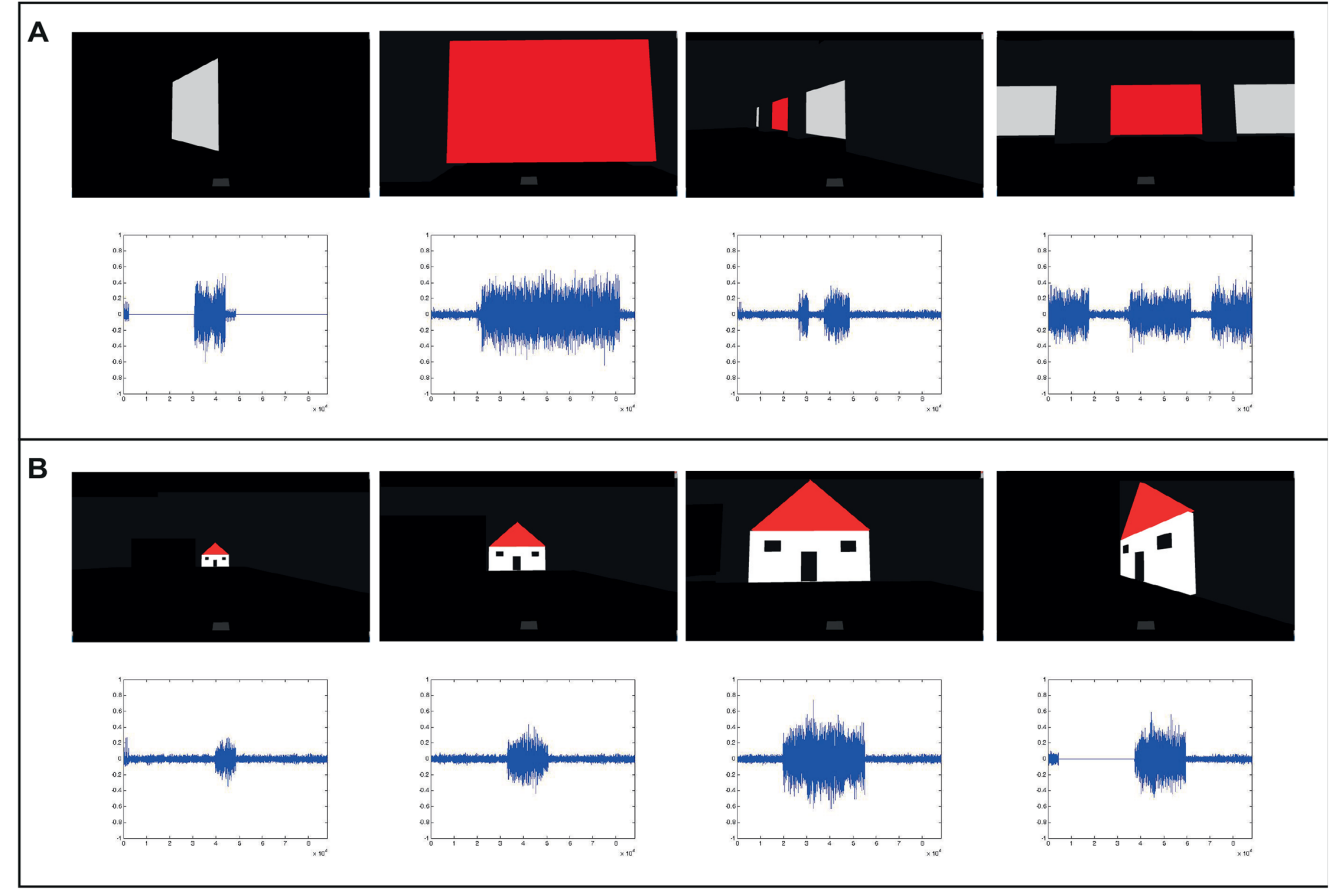
Auditory representation from different distances and angles. A demonstration of how the same scene or object can have very different representations both visually (shown here as screen shots) and auditorily (shown here as the corresponding EyeMusic waveforms) from different distances and angles, creating a significant challenge to maintaining object constancy and object identification. ((A) From Experiment1:task1, (B) From Experiment1:task2).

Participants experienced the objects in the scenes from different distances and angles, in which their visual representation and corresponding auditory representation were very different (Fig 6), making the task of maintaining object constancy and the mental map of their environment even harder. We suggest their successful results may be linked with the combination of active sensing, gamification and user immersion which have all been suggested in the past as potential SSD training boosters [19,22,30,44,45].

These dynamic differences also demonstrate that the users could not simply have memorized a short mapping of stimuli to succeed in tasks 1–2 and had indeed relied upon the EyeMusic transformation (Fig 6).

### Generic Use and Setup Availability

A key point of this approach is the generic option it offers for making existing virtual worlds accessible. Using SSDs to sonify the visual information presented on the screen does not require any pre-processing within the virtual environments, which has been a major stumbling block on previous accessibility attempts. Thus, a highly trained user should theoretically be able to navigate through any existing graphical virtual environment.

Additionally, our approach does not require dedicated hardware as we use only standard generally available interface tools such as a keyboard and headphones, increasing availability to users and circumventing the cost of expensive peripherals such as electrode or tactile arrays.

On the other hand, there are clearly advantages to the use of tools specially tailored for specific tasks, and the proper balance between the two approaches is under discussion [46]. We wish to clarify that the authors believe that the use of an environment-specific tool, when available, will currently probably lead to better results for tasks in the specific environment, though the new additional information offered via SSD is expected to be complementary to it. We do not see our approach as replacing these previous ones but rather as adding an additional tool to the accessibility toolbox for relevant situations.

### Complexity of the environments

In this experiment we mainly used very basic environments. Even our "complex" environment was relatively simple compared to mainstream virtual environments such as Google Street-view or World-of-Warcraft. Can these results extend to mainstream virtual worlds, which are far more complex and challenging? We suggest that with additional training the answer to this question is positive based on:

1. The ability of these same users, and many SSD users in general, to perform complex tasks with the EyeMusic in the real world which is even more challenging (see for example [28] in which experienced users used the EyeMusic to identify items in a noisy supermarket or [29,39] for examples from other SSDs).
2. Our subjective evaluation of the problems the blind users encountered, which in many cases were less in using the device but rather were focused on the concept of interaction with a virtual environment and visual rules, which we expect will improve with experience.

However it is clear that this must be explored in depth using more complex mainstream environments, and that the participants of this work will require more practice and training before they will be able to perform complex tasks in them.

### The contribution of virtual worlds to SSD training

The results shown here demonstrate that experienced SSD users can utilize their experience with SSDs in virtual environments, at least to a basic extent. This can work in both ways, enabling the use of virtual environments as a training platform for increasing skills in the real world. This is especially important as currently the main limiting factor for SSD use in the real world is the need for extensive one-on-one training and the lack of access to such programs by most potential users [14]. Online versions of such environments which are tailored to teach specific visual categories or concepts can be created, offering a much more complex tool for learning visual principles and SSD training than currently available and replacing at least some of this one-on-one training time while boosting the effectiveness of sessions with a sighted instructor.

Another key aspect is the ability to isolate skill-training–when using SSDs in the real world congenitally blind users must learn simultaneously to move through the worlds and aim the SSDs camera while at the same time learning visual principles and concepts and what different objects look like. Using virtual worlds, these features can be integrated in incremental steps enabling the user to tackle them one at a time. Though many skills will likely still require real world training, we expect the process to be easier and more successful after virtual training.

It should be emphasized that these environments are not expected to replace the current Orientation and Mobility training and instructors, but rather to augment them by giving instructors more tools, enabling more complex scenarios, enabling better tracking of user progress and enabling more efficient "homework" sessions between sessions with their instructors.

Future environments which are more realistic, such as using a Kinect-like 3D interface for moving and aiming the SSD, are expected to further increase immersion and learning.

### Use for the sighted population

The results from the sighted-blindfolded groups of experiment 2 demonstrated these tasks cannot be accomplished without vision, but that even minimal SSD training could enable some significant success.

The failure of the device-off group is unsurprising and in a way "unfair", but this demonstrates the exact problem–without assistive technology these environments are simply not accessible. They also suggest such tasks may improve the understanding within the sighted population of the need for accessibility.

Additionally, we described here the use of these environments for people with impaired visual input, but SSDs can also potentially add an additional multisensory dimension from a different perspective to the one usually used in such interfaces to the sighted as well. For example, using input from other SSDs sighted users could interact with dedicated virtual environments without the need for a large physical screen, or use this approach for purposes such as augmenting reality with additional information (as demonstrated in [47] for an augmented radiation sense, [48] for an augmented magnetic sense and some future suggestions in [30,42]).

### Color

Another potentially important factor, which influenced our choice of the EyeMusic SSD for this experiment, was the successful use of color information, which is a parameter the congenitally blind individuals completely lack any experience or perception of, and which has been shown in the past in the context of computer vision to boost scene segmentation when using degraded input [49] and in the context of SSDs to increase acuity [50]. Accordingly, this factor is increasingly being added into other new SSDs as well in various ways [51,52].

It should be noted that the colors used in this experiment were "standard" colors matching the discrete color categories of the EyeMusic. In environments with more complex color the EyeMusic will down-sample the colors by clustering them into one of its 6 colors. Future iterations of the EyeMusic, and other SSDs in general, should strive to grant the user access to a more complete color spectrum to avoid the loss of this information.

### Image processing and simplification

In this experiment the images presented by the EyeMusic were only naively down-sampled without additional pre-processing such as object recognition, filters, zooming or scene simplifications. These types of algorithmic modules are being integrated more and more into new SSDs and hold the potential to significantly boost users' perception, and indeed several are in the process of integration and testing with the EyeMusic [30]. Such processing can still be performed on the captured images from the screen without interfering with the genericity aspect as it does not require access to the environment's own code. In the current tasks users were able to perform successfully without them, but we suggest that in more complex environments such modules might be critical, and plan to use them in future work.

## Future Work

We have given here a proof-of-concept for this approach and demonstrated positive user reactions, but it is still far from being a practical tool for complex environments. Our best users will still require more time and training to be able to deal with such tasks, and a proper training program for it is currently being developed with the users. To further test this approach, and especially its practical limits, it will be important to use more difficult environments and larger groups of participants and compare the use of different existing SSDs conveying various visual features.

Another important aspect is the type of questions explored by this study. In this initial work we focused on perception and on basic navigation and did not address important issues such as memory and complex spatial cognition which are critical to the full use of virtual environments and should be addressed in future work.

An additional important potential use for this tool is for psychophysical and neuroimaging research, as it enables us to explore capabilities of blind individuals to perform tasks which are otherwise not possible such as landmark based navigation, complex multisensory interaction, or complex tasks interacting with a full environment. Importantly, these experiments can be performed within an fMRI scanner, and we are currently in the process of utilizing this approach to explore the neural correlates of space perception in the blind population.

## Conclusions

In conclusion, we have demonstrated here the potential of using sensory-substitution devices for increasing the accessibility of graphical virtual environments to blind individuals, and that participants were able to perceive the virtual environments and interact within them. Thus, this approach adds another tool to the accessibility toolbox for non-visual interaction with graphical virtual environments. This in turn enables such environments to be used as tools for SSD training, cross-device visual rehabilitation training and for safely learning new real world environments virtually before visiting them physically.

## Supporting Information

S1 Fig The stimuli used in the warm-up session. Note that not all users experienced all stimuli.
(EPS)

S1 File EyeMusic Warm-up and training session. (DOCX)
