# Supplementary material for: Perception of Graphical Virtual Environments by Blind Users via Sensory Substitution
Source: PLoS One. 2016 Feb 16;11(2):e0147501. doi: 10.1371/journal.pone.0147501 (PMC4755598; doi:10.1371/journal.pone.0147501)
Supplement: S1 File — (DOCX) [file pone.0147501.s002.docx]

Supplementary text 1 – EyeMusic Warm-up and training session

For the blind participants of experiment1, training consisted of a series of static "warm-up" stimuli followed by a reminder discussion of visual principles and making sure they understood the keyboard controls. There were no official pre-tests, beyond asking them to turn left, right and then walk forward in an empty virtual room to verify key-strokes. The experiment was started when users felt confident enough to begin.

For the sighted participants of experiment 2 we used the basic EyeMusic training stimuli in an adapted version of the regular training session (see stimuli in S1 Fig, see protocol description in [32]). The experiment was started when users felt confident enough to begin.

Then the following list of visual principles was discussed in depth with the participants:

1. The effect of turning on the visual field
2. The effect of changes in angles on a target
3. The effect of changing distance on the visual field
4. The effect of changing distance on a target
5. Occlusion between objects
6. Relative distance between objects
